# Supplementary material for: Molecular insight into RNA polymerase I promoter recognition and promoter melting
Source: Nat Commun. 2019 Dec 5;10:5543. doi: 10.1038/s41467-019-13510-w (PMC6895186; doi:10.1038/s41467-019-13510-w)
Supplement: Supplementary file 1 — Supplementary Information [file 41467_2019_13510_MOESM1_ESM.pdf]

Supplementary Information

Molecular insight into RNA polymerase I promoter recognition and promoter melting

Sadian et al.

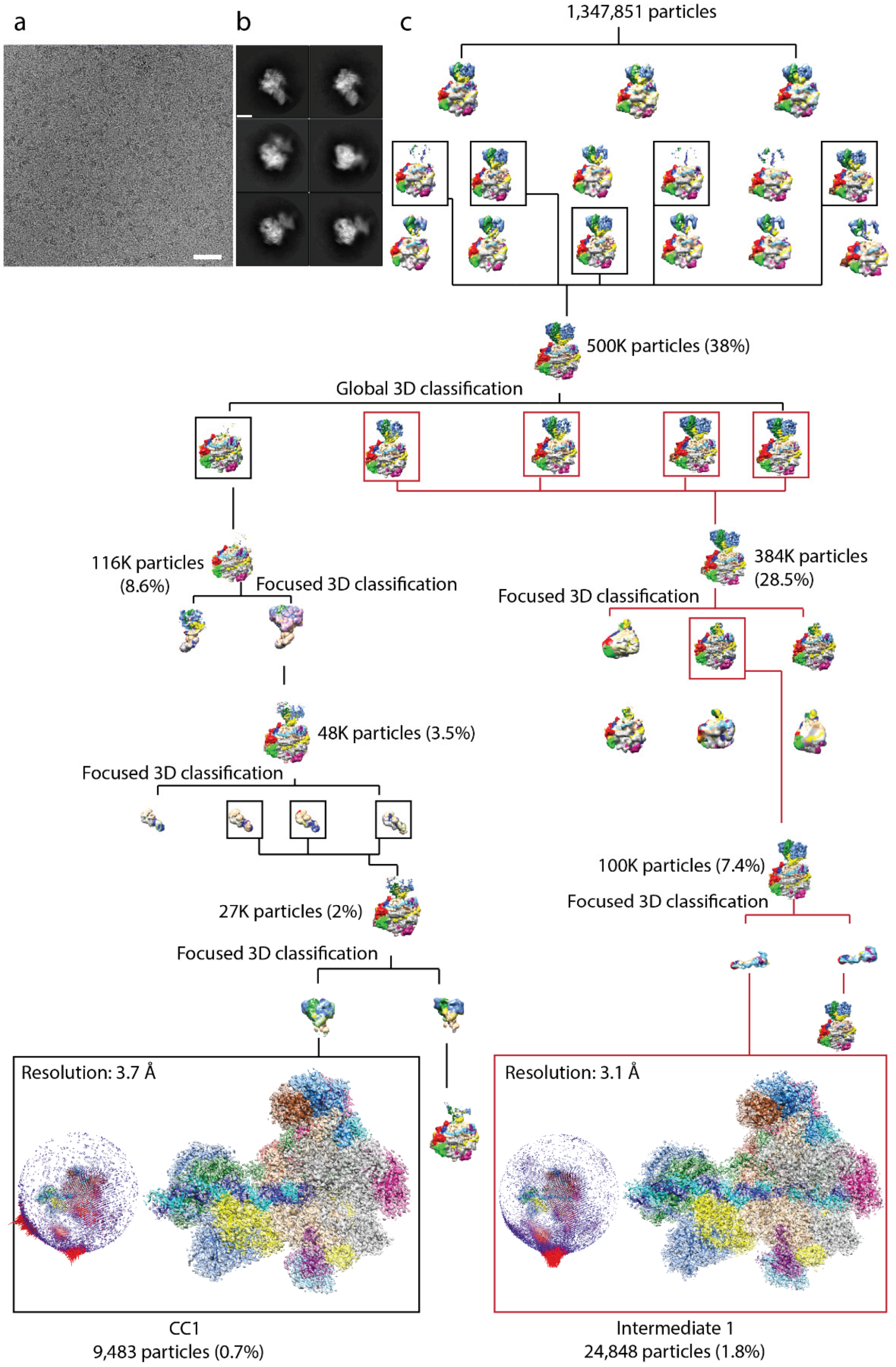

Supplementary Figure 1. Processing of the CC1 dataset. (a) Representative micrograph of Pol I CC1 dataset. Scale bar is 50 nm. **(b)** Representative 2D class averages of Pol I CC1 dataset. Scale bar is 10 nm. **(c)** 1,347,851 particles were initially picked using low-pass filtered Pol I PIC and Pol I EC 2D class averages as a reference[1]. The particles were 2D sorted and divided into three independent groups after 2D sorting. Each class was separately refined and classified. The classes that contained the highest number of particles or showed high resolution (boxed in black) were pooled and classified further. Class 1 (boxed black) was separately classified for a few rounds using a mask on CF, DNA and CF-DNA densities and finally 9,483 particles were sorted to the Pol I CC1 class. Other classes (boxed red) were pooled and classified using a mask on the Pol I-Rrn3 and the heterodimer (A49/A34.5) densities to obtain the Pol I Intermediate 1 class (24,848 particles). Pol I CC1 and Pol I Intermediate 1 densities (with the fitted models) and the corresponding angular distribution are shown in large black and red boxes respectively where all subunits are coloured according to the colour code in reference [1].

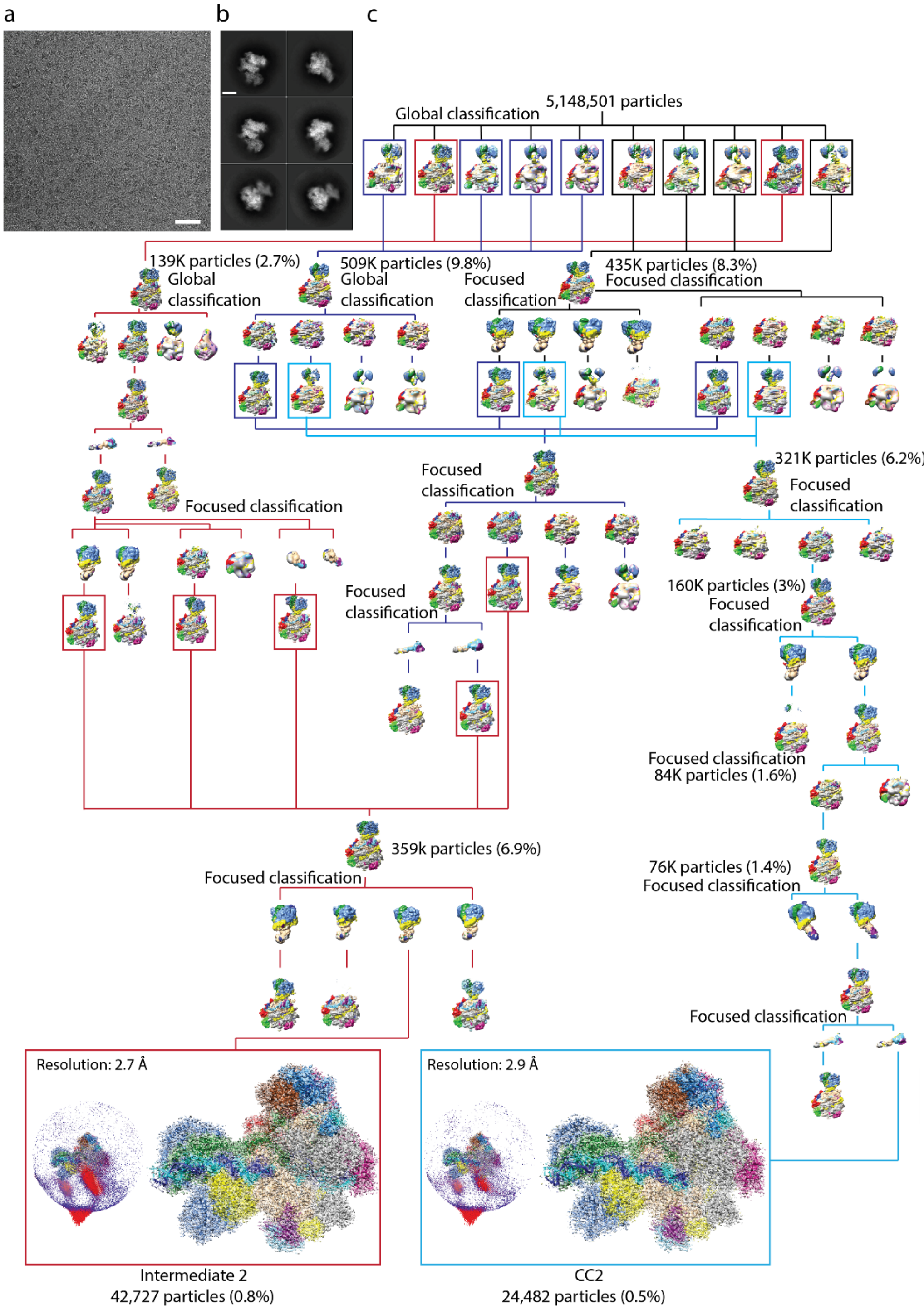

Supplementary Figure 2. Processing of the CC2 dataset. (a) Representative micrograph of Pol I CC2 dataset. Scale bar is 50 nm. **(b)** Representative 2D class averages of Pol I CC2 dataset. Scale bar is 10 nm. **(c)** 5,148,501 particles were picked, 2D sorted, divided into eight groups and classified. Two classes (139,819 particles) that showed high-resolution features (red box) were pooled and classified independently. In parallel, other classes (508,985 and 435,520 particles) were divided into two groups (dark blue and black boxes) and classified separately. Particles that showed a density for an open DNA and high resolution for CF (dark blue) were pooled and classified using a mask on the Pol I-Rrn3 density. The first class was classified with mask on the heterodimer density that together with the second class of the previous classification were pooled with particles boxed in red and classified using a mask on the CF and finally 42,727 particles were sorted into the Pol I Intermediate 2 class. Particles that showed a density for both downstream and upstream dsDNA were pooled (boxed cyan) and classified with a mask on Pol I-Rrn3, CF, Pol I-Rrn3-DNA, CF-DNA and heterodimer to get to the final class of Pol I CC2 (24,482 particles). Pol I CC2 and Pol I Intermediate 2 densities (with the fitted models) and the corresponding angular distribution are shown in large cyan and red boxes where all subunits are coloured according to the colour code in reference [1].

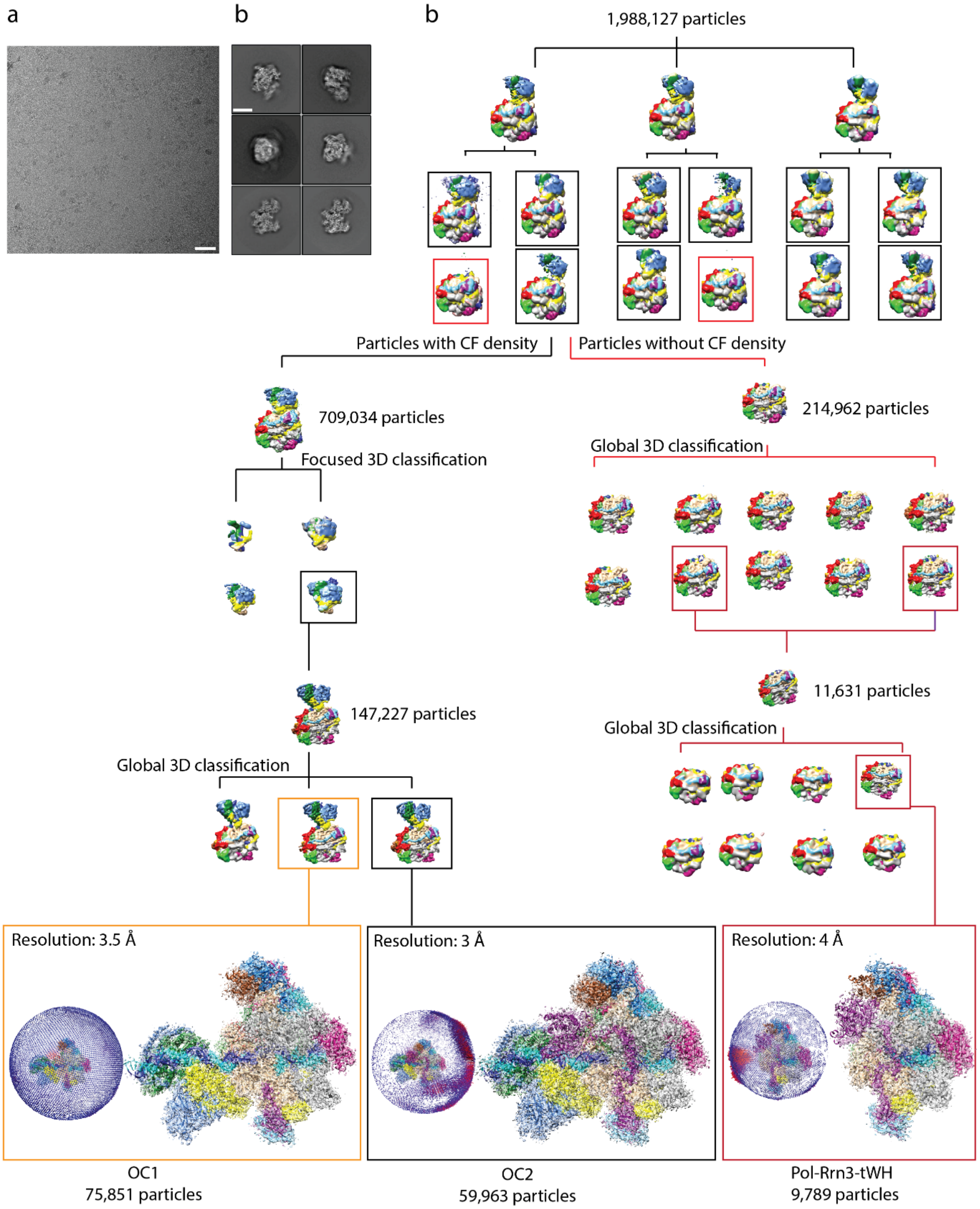

Supplementary Figure 3. Processing of the artificially induced OC dataset. (a) Representative micrograph of Pol I OC dataset. Scale bar is 50 nm. **(b)** Representative 2D class averages of Pol I OC dataset. Scale bar is 10 nm. **(c)** 1,988,127 particles were picked, 2D sorted and divided into three groups that each was separately refined and classified. The classes that showed a density for CF (709,034 particles) (black box) or no density for CF (214,962 particles) (red box) were pooled and classified separately. The particles with a CF density were classified using a mask on the CF density and further globally classified to obtain the Pol I OC1 (75,851 particles, orange box) and the Pol I OC2 (59,963 particles, black box). The particles without a CF density were classified and the classes that had a density for tWH were separately refined to obtain a reconstruction for Pol-Rrn3-tWH (9,789 particles, red box). Pol I OC1, Pol I OC2 and Pol-Rrn3-tWH densities (with the fitted models) and the corresponding angular distribution are shown and coloured as in reference [1].

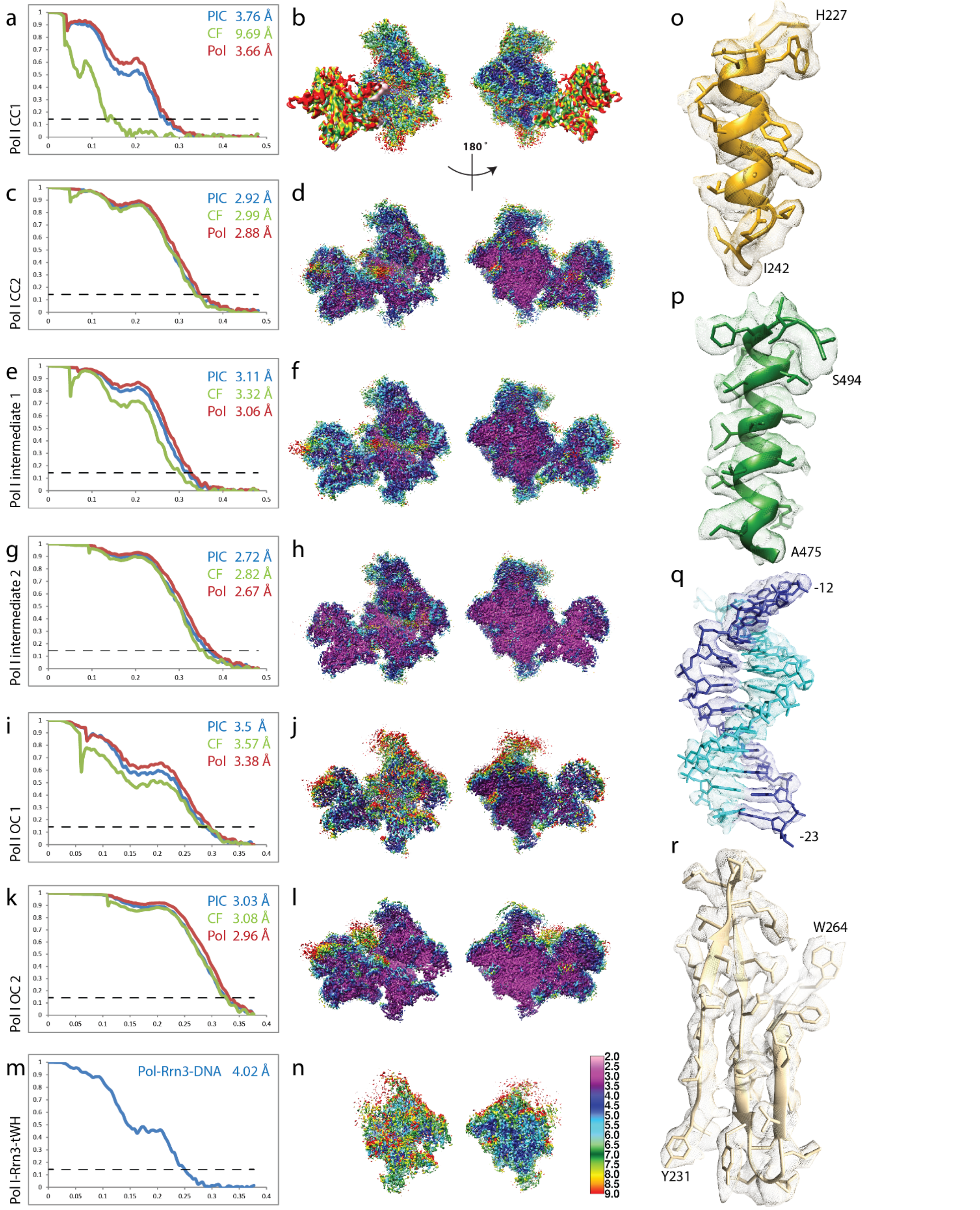

Supplementary Figure 4. Cryo-EM of Pol I IC datasets. (a), **(c)**, **(e)**, **(g)**, **(i)**, **(k)** and **(m)** Fourier shell correlation curves of the represented reconstructions of Pol I CC1, Pol I CC2, Pol I Intermediate 1, Pol I Intermediate 2, Pol I OC1, Pol I OC2 and Pol I-Rrn3-tWH respectively. Blue, green and red lines correspond to global refinement, CF and Pol-Rrn3 focused refinements respectively. The black dotted line indicates the 0.143 criterion. **(b)**, **(d)**, **(f)**, **(h)**, **(j)**, **(l)** and **(n)** Local resolution of the multibody refined Pol-Rrn3 and CF maps (Left: front view. Right: back view). The colour bar indicates the resolution range used for colouring the maps (2-9 Å). (**o**) Model and density of Pol I OC intermediate 2, Rrn11, residues 227-242. (**p**) Model and density of Pol I OC intermediate 2, Rrn7, residues 475-494. (**q**) Model and density of Pol I OC intermediate 2, upstream DNA -12 to -23. (**r**) Model and density of Pol I OC intermediate 2, A135, residues 231-264. The models are coloured as in reference [1].

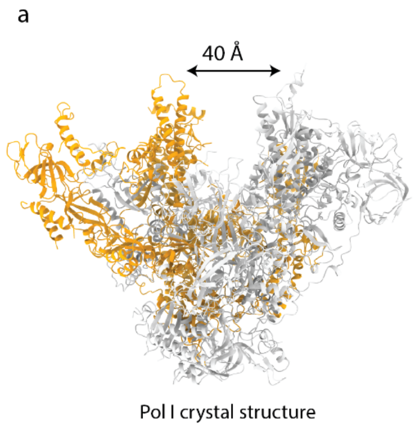

Supplementary Figure 5. Comparison of cleft width of Pol I CC2. (a) Pol I crystal structure (PDB: 4C3I), **(b)** Pol I-Rrn3 structure (PDB: 5G5I), and **(c)** Pol I CC2 are represented in grey ribbons except A190 coloured in orange, dark green and light blue in a, b and c respectively. Rrn3 is coloured in dark khaki and brown in Pol I-Rrn3 and Pol I CC2 respectively. Pol I CC2 DNA is shown in dark blue. The cleft width is measured between Cα atoms of A190 residue 429 and A135 residue 423.

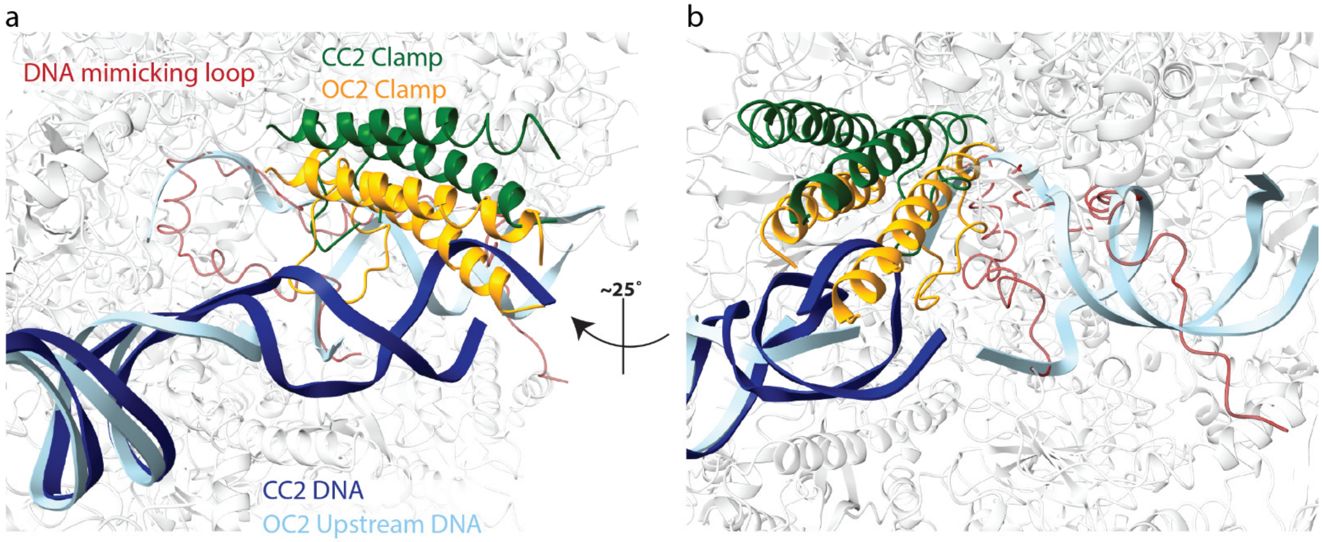

Supplementary Figure 6. DNA mimicking loop of Pol I CC2 and Pol I OC template strand occupy the same position. (a) and **(b)** Pol I CC2 is shown in grey ribbon presentation with the DNA mimicking loop of A190 (residues 1350-1420) in red. The clamp coiled-coil helices (A190, residues 380-460) of Pol I CC2 and Pol I OC2 are coloured dark green and light orange respectively. The DNA of the Pol I CC2 and Pol I OC2 are coloured in dark and light blue respectively. The DNA surrounding the TSS in the Pol I CC2 clashes with the clamp position in the Pol I OC2, while the DNA in the Pol I OC2 displaces the DNA-mimicking loop present in the Pol I CC2.

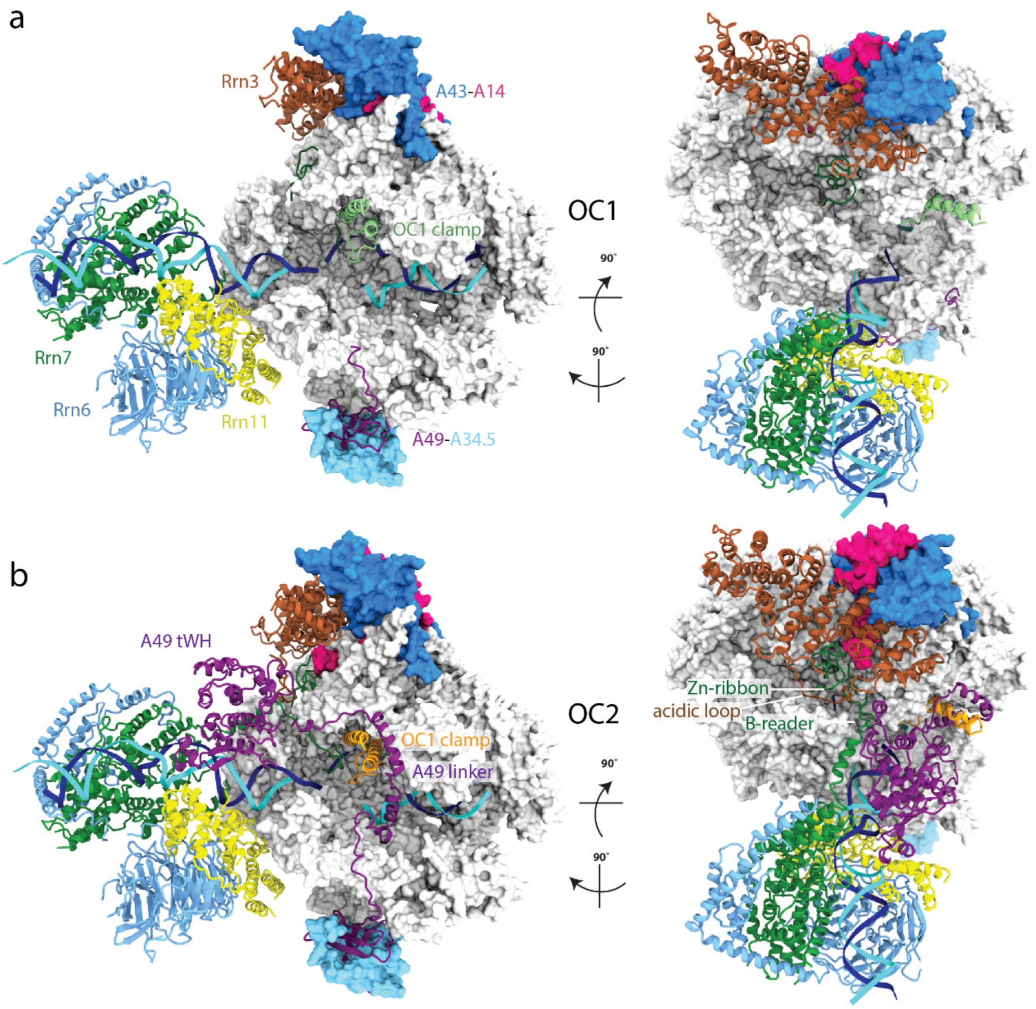

Supplementary Figure 7. Comparison between Pol I OC1 and Pol OC2. Side (left) and top (right) views of **(a)** Pol I OC1 and **(b)** Pol I OC2 are shown and coloured as in Fig. 2 except A190 clamp coiled-coil helices depicted in light green and light orange in Pol I OC1 and Pol I OC2 respectively. The clamp coiled-coil is partially closed in Pol I OC2 compared to Pol I OC1 that allows wrapping of the A49 linker and therefore positioning of the A49 tWH next to the promoter. B-finger and Rrn3 acidic loop are disordered in the Pol I OC1.

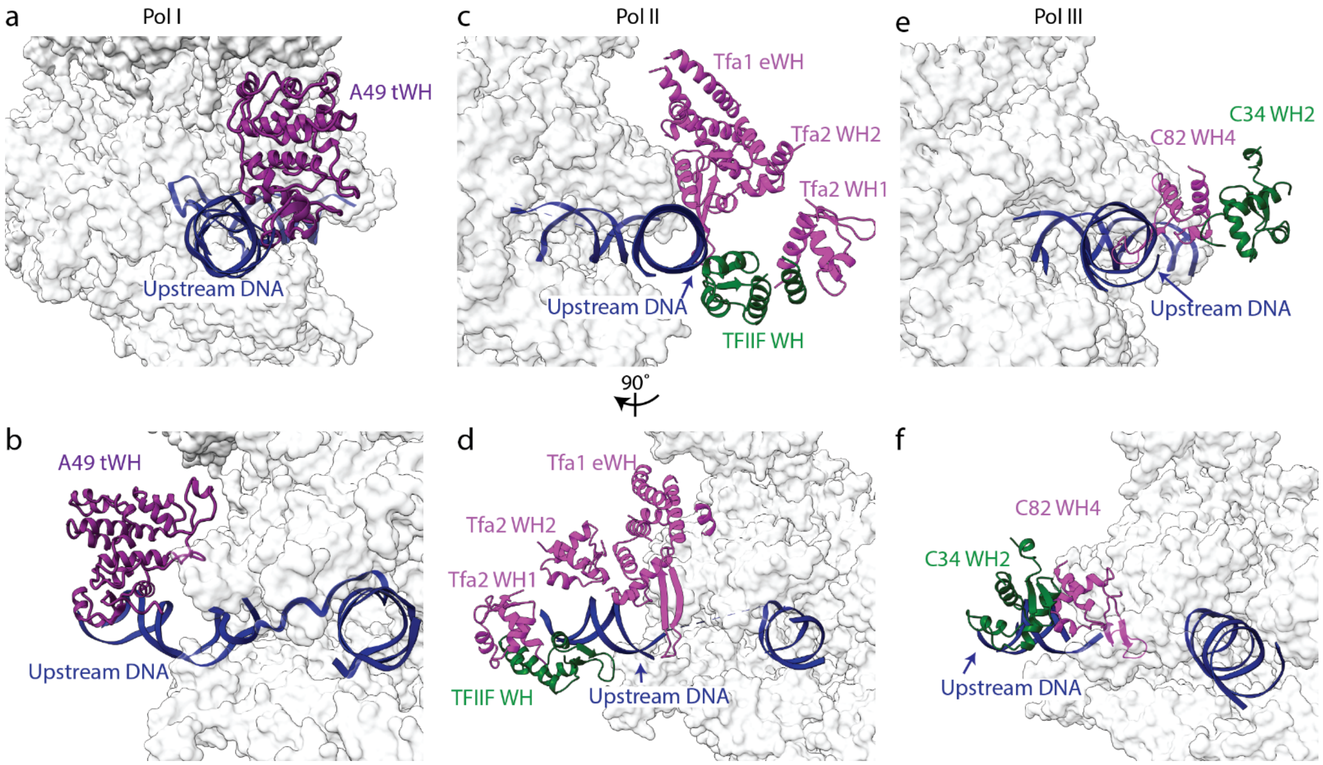

Supplementary Figure 8. Comparison of Pol II TFIIE and TFIIF with Pol I and Pol III homologues. **(a)** and **(b)** Pol I OC2 is shown in grey surface representation with the A49 tWH and the DNA in purple and dark blue ribbon, respectively. **(c)** and **(d)** The A49 tWH occupies a similar position as TFIIEβ (Tfa2) in the Pol II OC (PDB: 5FYW). **(e)** and **(f)** In the Pol III OC (PDB: 6F40), C82 WH4 and C34 WH2 resemble Pol II OC Tfa1 eWH and TFIIF WH, respectively, but a similar structural homologue in the Pol I OC is not present. **(b)**, **(d)** and **(f)** show 90° rotated view with respect to **(a)**, **(c)** and **(e)**.

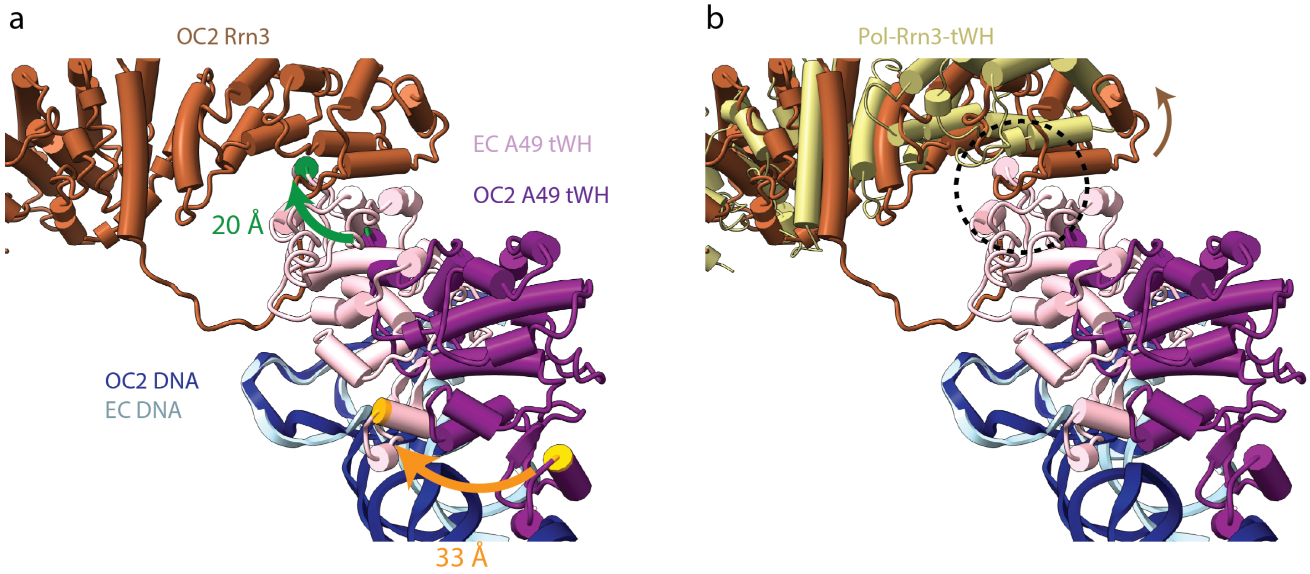

Supplementary Figure 9. A49 tWH clashes with Rrn3 in the EC position. (a) The position of the A49 tWH of the Pol I OC2 and Pol I EC (PDB: 5M64) are compared. A49 tWH of Pol I OC2 and Pol I EC are shown in purple and pink ribbon respectively. Rrn3 of Pol I OC2 is shown in brown as tubes. The residues 314 and 382 of A49 are coloured in green and orange respectively to indicate the rotation required to transition between the OC and EC positions. **(b)** Pol I OC2 is superimposed on Pol I-Rrn3-tWH structure obtained from the OC dataset. The A49 tWH of Pol I OC2 and Pol I EC are coloured in dark purple and light pink ribbon respectively. Rrn3 of Pol I OC2 and Pol I EC are coloured in brown and dark khaki respectively. The clash between A49 tWH in the EC position (similar in the Pol I-Rrn3-tWH structure) and Rrn3 is depicted in dashed circle. Because of the transition from the OC to EC, Rrn3 is pushed back slightly by the A49 tWH. All structures are aligned on A135.

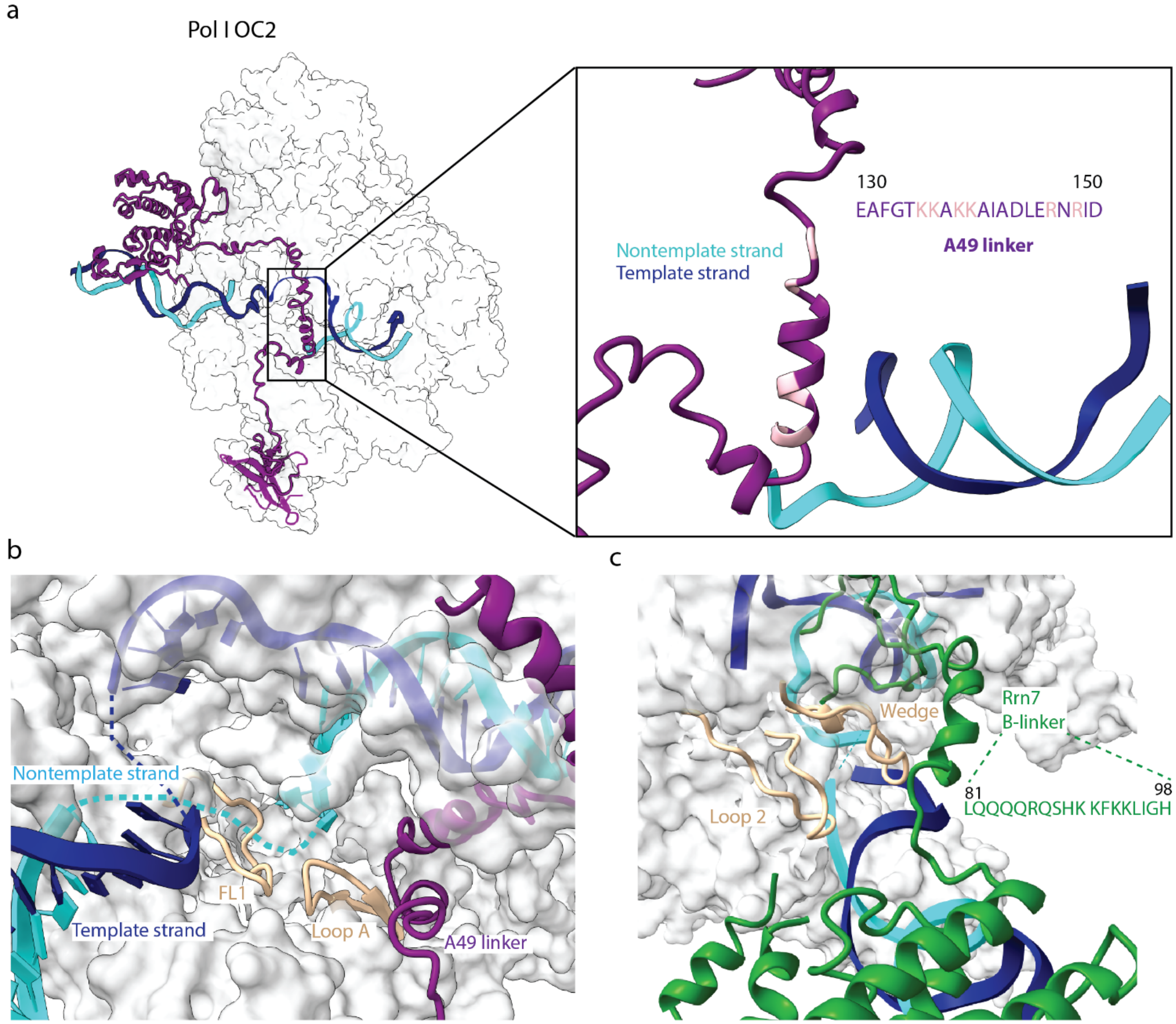

Supplementary Figure 10. Transcription bubble stabilization. (a) Pol I OC2 is depicted in grey surface with the A49, template and non-template strands in dark purple, dark blue and cyan ribbons respectively. In the right panel, a zoom-in view of the A49 linker helix is shown where basic residues of the linker helix (residues 130-150) are coloured in light pink. The basic residues are positioned close to the transcription bubble and as a result stabilize the DNA in the cleft. **(b)** The putative path of the non-template strand is depicted in a dotted cyan line, which goes through the Fork Loop 1 (FL1) (A135, residues 470-484) and the loop A (A135, residues 260-271). The A49 linker further constraints the non-template strand in the Pol I cleft. FL1 and Loop A are shown in wheat ribbon and the A49 is coloured in purple. **(c)** The upstream edge of the transcription bubble is stabilized by the wedge (A135, residues 810-826), Loop2 (A135, residues 885-900) and Rrn7 B-linker helix (residues 81-98). Wedge and Loop2 are coloured in wheat and Rrn7 in green ribbon.

CF Rrn6

ATGTCCGAAGGTCAAATCCCGAGCAGCGATGTTCTGGGTAGCCAACTGGGCGTGGGTGTTCAAGGTGCCTCCCTGTATTGTCCGCAGGAAAACTA
CACCACCAAGAAACAGGAGAAGCCGCAATGGCTGCGTCCGGTGGATGATACCCTGGCGGAGGATGCTCTGGACCTGCACATCGTGGTTAAGAGC
CTGCTGTGCGATACCGCGATCCGTTACATCAGCGACGATAAAGTTCTGCAGGAAAGCGACGCTGACGATGACCTGATCACCAGCGATATCGACGA
GGATACCGACAACCAGGGTGACACCAGCATCGTGGTTAACCCGGTTATCCCGGTGGTTCCGAAGGACGTTCACTTCTTCAAGAAAGTGGATGTTG
GTAACGACAGCATGTTCGGCGTGAACTGCGACACCCCGGTTAGCTTCCAGGATTACATCCCGAGCGACCTGCTGCGTAACCTGGATGACACCCTG
CAGGAAAGCACCAACAGCAGCCGTCCGATGCAGGATGCGTTCTTCTGGGACCCGACCGTGGCTAACCGTCTGGACAGCCAGTACATCCAGACCGC
GAGCGATCTGCGTAACTACCGTGACGGTACCGAGATCATCGCTTACGCGAGCGGCAAGACCGGCAGCGTGCTGAACATCGCTGTTCTGACCCGTC
AGAACACCCTGCACCTGAACCGTCACAACAACGTGACCAGCATCGAACTGCACAGCCCGATCAAGAGCATCAAAATTCCGGGTGCGAGCGAGAG
CATCGGTCGTCGTAGCAACCTGGTGGGCATCATCACCGAAAACAGCTTCCAGATCTTCCGTATCGAGAGCGTTCACAGCCGTAGCTGCGACGTGA
TGGTGAGCAGCAGCGAACCGCTGTACTTCGTGGAGATCGATGACCTGCAGGTGGTTGATTTCGCGTTCAACCCGTGGGACCTGCAGCAGTTCGCT
ATCATCGACATCAAGGGTAACTGGAGCATCGGCCGTATCCCGAAAAACTTCAACAACAACAACAAGCGTAAACTGCAGCTGATCGATAACCTGCA
CGGTACCATCTTCGACCCGGAGGAACTGAGCAGCTGGAAGCGTATCGAGTGGTTCAGCCACTTCCAGAAGATCCTGGTGTTCGATCGTAGCAAAA
TGATCGAAATCGACTTCATGAACAACTGGCAGACCGAGGTGGTTCAGGCGAAGGCTTGGAGCAACATCCGTGACTACAAGCGTATCGATGACAA
AAACGGTATCCTGCTGACCAGCCGTGAAATCATCATCGTTGGTGCGAGCGAGAGCAACGACCCGGTTCGTCGTATCAGCTGGAAGCACGATCTGG
ACCCGGATGACACCACCCTGCGTATCACCGTGCAGAAAGTTAAGAAACCGGACCACATCCTGCTGGTTGCGTTCGTTTACAGCATGCGTCACAAG
CGTATCTACATGCACGTGTTCAGCCACCGTAAAGCTAACCTGTTCCAAAGCCTGGGTTGCAGCACCGTTCTGGAAATTCCGGGTGGTACCCCGACC
GGCATCGAAACCATCCTGACCCTGGATCACATCGATGACGAAAGCCGTCGTGAGGAAGATGCGGACGAAAACTTCGAACTGGTGGTGGACTTCCT
GGTGAAGCTGCGTAACAGCAGCGAGGTTTACTACTACGCTCTGAGCAACACCCAGAACAGCGAACCGAACAAACAGGAAACCCCGATCATCGTG
GATCACCCGGAATGGGCGAGCCTGTTCAACAACGCTGACGAGCGTGAAAAGGAGAGCATCGGTGCTCTGGTGAGCCAGATCAAGCTGAAAGAAC
GTGAGCGTATCAGCCGTGTTCAAAACCTGATCGAACACGAGAACAGCCACGATGAAGACAAATACCTGCAAGACCTGGGTTACCGTCTGAGCATC
GCGACCAACGAACTGCTGGAGAGCTGGCAGAAGACCAAAGACGAGAGCATCCTGAGCGGCAGCCTGAGCCACAGCAAGCTGAAAAACCTGCTG
GAAAACAGCGATAGCTTCGCGAGCATCCCGGAGTTCAGCAGCCTGCTGGACCAGTTCTTCCAGTACTACCAGGATCAGGACGTGACCTTCATCGG
TTTCGAAAAGCTGCTGCACCTGTTCCTGCACGAGGATGTTCCGGGCCTGGACATCTTCTACAACAAACTGCTGCAGTGCTGGGTGCTGGTTAGCCC
GCAGGCGGAACTGCTGACCAAGGAGATCGTGAAAGACATCATCTGGAGCCTGGCTCGTCTGGAGAAGCCGAGCCTGTTCGAACCGATCCAGAAC
GAGATCAGCCGTAGCCTGAGCGGCCCGTACCAGGATATCATTAGCAGCTGGGACATGGATGACATCAACGAGGAAGATGAAAGCAACGAGTTCA
ACTTCGACAGCCAGTTCAGCGCGCCGTTCAACGGTCGTCCGCCGTTCAACCTGAACAGCCAGAGCCAGATCCCGACCATCAAGAGCAGCCAGAGC
AGCGGCCTGGCGCGTCGTAAGCGTATCCTGAAAACCCAGAGCCAGAAAGCTACCCCGCTGAGCCAGAGCACCCAAAACCTGAGCGTGCTGCCGG
ATAGCATGACCCCGGCTTTCACCCTGATGCAGCCGCCGAGCAGCCAGATCAGCTTCGTTAACGACAGCCAACCGCGTAACTCCCAAAAGGCAAAG
AAGAAGAAGAAGCGTATTCGTGGTTTCGGCTA

CF Rrn7

ATGAGCACCTTTATTCGTGGTCCGATCTGTGGCACCGACAACTGCCCGTCCCGTCTGTGGCGTATTATTGATGGCCGTCGCACCTGTCAGTACGGC
CACGTGATGGAAGGTGACGTTGAGTTCAACGACGATGAGGACGATCTGAACGGTCTGGGCGCTGGTGTGATCACCCGTCGTCTGAACCTGACCAC
CAACGCGACCGGCAGCTTCCAGAGCAGCCAGCTGACCAACAGCCAACTGCTGCAGCAGCAGCAGCGTCAGAGCCACAAGAAATTCAAGAAACTG
ATCGGTCACGAAGCTAAGCTGCTGTTCCTGAAAAGCTTCCAGTTCATCCTGAAGCGTCAGATCCGTTGGCTGATCACCGAGATGCGTTTCCCGAAG
GAATTCGAGCACGTTGCGAAAATCATCTGGCTGAAGATCCTGAAAACCATCAACGACCAGCCGCAGGAAGAGCTGAAGCTGCAGCTGCACATGA
CCAGCACCATCAGCATCCTGTACCTGGCGAGCACCCACCTGAGCCTGCCGGTGTACACCTGCGATTACATCAAGTGGATCTGCACCGCTAAAATG
CCGTACTTCCAGGCGAGCGAAATCCTGCCGAAGAGCTGGCGTATCCAGCTGCCGAACTACTACGTTAGCATCCTGGAGGGCAGCATCAGCCCGTT
CAACGGTCAGCTGTACAACAAGATCGCTCTGACCTGCGGCATGATCCACTTCAAAGAATTCTTCAACAGCGAGATCAGCTGCCAAGGTCTGCTGC
TGAAGCTGGTTATGCAGTGCGCGCTGCCGCCGGAATTCTACTTCTACACCAAACAGGTTATCGAGTTCGAGGAAACCGACATCCGTAACCTGACC
CTGTGGGAACGTACCGATGAGCGTCACACCGGCCGTGTGAGCAACCACGCGGAACTGCGTGTTCTGAGCTACTTCATGCTGACCATCAACTGGAT
GCTGAGCTTCGACCGTGATCGTCAGTACCCGCTGAAGTGGATCCTGAGCCTGACCGAAAGCCTGACCCAGCGTACCACCACCAGCGAGAGCATCG
GTCGTAACATCGTGAAGGTGGTTTACCCGGACAAACCGACCAGCAGCGATTACTTCCAGTGGAGCGAGGAAGAAACCCTGGAATTCCTGAAGTGG
ATGGAGAAACAATTCCTGCCGACCCAGACCAAGAGCCTGCACAACGAAAACGGCAGCATGGAGATGACCATCGACCAGAAAATCGCTCGTCGTA
AGCTGTACAAAATCTTCCCGCTGGATCGTGAAGCGAACCACGACGGCGAGTTCAACGATAGCACCCACCAGCTGACCTTCATCGAAGACCTGCAG
GAGCGTTACGCTAAGCAGACCCCGTTCTTCGAGAGCAACAAAATCCGTGATAGCCTGAACTACCAGGAAGCGAACCCGCCGGCTCGTAAGGAAG
CGATCGGCCGTCTGCTGACCCACATCGCGAGCCAACTGCTGGTTGACTTCGCGATCAGCAAAGAGCAACTGAAGGACTGTATCTCCCGCATCAAA
AACGCCTGCCTGCATCGTATGAACTAA

CF Rrn11

ATGTTTGAAGTGCCGATTACCCTGACCAACCGCAAGTTCGCCCAGCGTCGCAAGCTGAAATACCAATACATTAACTACATCTCCCGTCGTTTCGAT
CGTATCAGCAAGAAAAGCACCACCACCGATAGCCTGCCGACCCCGGAGAACAGCGCGGCTGAAAACAACGATGAGGAAGAGGGTCAGAACAGC
GAAGCTGGCACCTACCGTCGTAGCGTGCTGCAGCAGAAGAAGCGTCGTCGTGAACGTCACTGGCGTAGCGTGGTTGGCGAGATCTACAGCACCAC
CGAGAGCGAAACCGACAGCCAGGAAGAGGAAACCGAGGAAGGTGGCGAACACGACACCGGCATCGATAAGGAAGACAGCGATGAGGAACGTA
AATTCTGGAAGAAATACGAGAAGCCGGAGAAGAGCTTCGAAATCTGGCGTACCGTTAGCAGCCAGAACAAGCAGCCGATCAACAAGCAGAAAAT
GACCTACCACAACTTCAAGAAAATCGAAAAGATCCCGCTGCGTAAAATGGAGATCCCGCTGCTGCACTGCACCAAGGAGAACAAACTGTACTTCC
AGAGCATCAGCCGTGGTCTGGAACCGCTGAAGACCAGCACCAGCGAGGTGCGTAACTACCGTACCCGTCACATCGTTACCCTGACCGACCTGCTG
CACCTGAACGTGAGCCGTCACAACTGGAGCCTGGCGTACAAGATCTTCGCTACCCTGATCCGTATTCCGGGTGTGCAGATCAAAAGCCTGTGGGG
TATCGGCGTTGAGATCCTGGATAACCTGAGCAACAGCAGCAGCGGCCTGGACTTCCTGCAGTGGATGTGCCAGATCTACAGCAGCAAGAGCCGTT
TCGTGCAGAACATCAACTACCGTAGCATCGTTCCGCCGTTCCAGACCGGTAGCCGTACCCACACCGCGAAATTCGCTATCACCTACCTGTGGAGCA
GCCTGATCAACTGCCAGAAGAGCATGGAACCGAGCAGCAACATCATCGACAAACCGTTCGATACCGAGAACGACCTGCTGCAAGAGCTGATCGA
TAAGATCAGCGAGTGGGTGCTGACCCCGCCGTTCATGGAGGACGCGGAAGTTTGGTTCATCTACGCGAGCTGCCACCTGCTGAAGGCGGATACCC
TGAGCCGTCAGTTCGTGAACGATAACAAAAACAACGACCTGATCGGCCTGGACCGTGATATCAAGATCAACCAGGTTATCAAGCACATCCACTAC
GTTCGTACCTTCCTGAAGATCTGCCTGGACAAAGGTGGCTTCGCTGTTCCGAGCCGTCTGATCGAAAACCAGCTGAAGAGCTTCGAGAGCCGTCTG
TACGGTGAAGCTCAGGATATCCAGGAGCGTGACGTGGCGAACGTTTACGACAGCATCGATAACAGCAGCGTGGAGAACAGCTTCGGCGACGTTT
ACGAAACCAACGCGGAATTCCTGGATACCCAGCTGATGGACCTGTCCCCGGAAGATAACGGTCTGGACGAAATGCACTACAGCGACGAAGATAG
CAGCGAATAA

Supplementary Figure 11. Codon optimized sequences for *Saccharomyces cerevisiae* CF subunits Rrn6, Rrn7 and Rrn11.

Supplementary References

1. Sadian, Y. et al. Structural insights into transcription initiation by yeast RNA polymerase I. *EMBO J* **36**, 2698-2709 (2017).
