# Supplementary material for: Molecular insight into RNA polymerase I promoter recognition and promoter melting
Source: Nat Commun. 2019 Dec 5;10:5543. doi: 10.1038/s41467-019-13510-w (PMC6895186; doi:10.1038/s41467-019-13510-w)
Supplement: Supplementary file 3 — Description of Additional Supplementary Files [file 41467_2019_13510_MOESM3_ESM.pdf]

Description of Additional Supplementary Files

Supplementary Movie 1: “RNA polymerase I promoter opening. The movie shows the Pol I promoter opening that includes several transitions from CC1 to OC2 as also depicted in Figure 6.”
